# The manipulation of strain, when stress is controlled, modulates in vivo tendon mechanical properties but not systemic TGF-β1 levels

**DOI:** 10.1002/phy2.91

**Published:** 2013-09-23

**Authors:** Gerard E McMahon, Christopher I Morse, Adrian Burden, Keith Winwood, Gladys L Onambélé-Pearson

**Affiliations:** 1 Department of Exercise and Sport Science, Institute for Performance Research, Centre for Lifespan and Wellbeing, Manchester Metropolitan UniversityCrewe Green Road, Crewe, CW1 5DU, United Kingdom; 2 Sports Institute Northern Ireland, University of UlsterShore Rd, Newtownabbey, BT37 0QB, United Kingdom

**Keywords:** Muscle–tendon complex, soft tissue biomechanics, stiffness, stress/strain, stretch

## Abstract

Modulators of loading-induced in vivo adaptations in muscle–tendon complex (MTC) mechanical properties remain unclear. Similarly contentious, is whether changes in MTC characteristics are associated with growth factor levels. Four groups were subjected to varying magnitudes of stress/strain: Group 1 trained with the MTC at a shortened position (MTCS; *n* = 10); Group 2 at a lengthened position (MTCL; *n* = 11; stress levels matched to MTCS); Group 3 over a wide range of motion (MTCX; *n* = 11); and Group 4 (*n* = 10) was the control population (no training). Patella tendon Stiffness (*P* < 0.001), Young's modulus, and quadriceps torque (*P* < 0.05) increments (only seen in the training groups), showed MTCL and MTCX groups responses to be superior to those of MTCS (*P* < 0.05). In addition, MTCL and MTCX better maintained adaptations compared to MTCS (*P* < 0.05) following detraining, with a pattern of slower loss of improvements at the early phase of detraining in all training groups. There were no significant changes (*P* > 0.05) in antagonist cocontraction, patella tendon dimensions or circulating transforming growth factor beta (TGF-β1) levels following training or detraining in any of the groups. We conclude that chronically loading the MTC in a relatively lengthened position (which involves greater strains) enhances its mechanical properties, more so than loading in a shortened position. This is true even after normalizing for internal stress. The underlying endocrine mechanisms do not appear to be mediated via TGF-β1, at least not at the systemic level. Our findings have implications with regard to the effectiveness of eccentric loading on improved tendon structural and mechanical properties.

## Introduction

Resistance exercise training is undertaken or prescribed for a variety of reasons including sports performance, aesthetics, general health, functional maintenance, recovery (e.g., following injury, illness/diseased states), and also to offset the effects of ageing. During resistance exercise, skeletal muscle and the in-series tendon (the muscle–tendon complex [MTC]) work together in a concerted effort to bring movement about a joint (Ker et al. 1988). The MTC displays high levels of metabolic activity following acute exercise (Miller et al. 2005; Bojsen-Møller et al. 2006; Kubo et al. 2008) and also plasticity following a prolonged training regime (Arampatzis et al. 2007a; Seynnes et al. 2009). The synchronization of tendon adaptation simultaneous to muscular adaptation is critical to preserve MTC function and to prevent injury. It is also therefore imperative to identify the mechanisms by which such function is modulated.

Following prolonged periods of resistance training, it has been demonstrated that tendon (e.g., patella and Achilles) has the ability to adapt both morphologically (Arampatzis et al. 2007a; Kongsgaard et al. 2007; Seynnes et al. 2009) and in terms of intrinsic material properties (i.e., increased stiffness and/or Young's modulus [Reeves et al. 2003; Maganaris et al. 2004; Kubo et al. 2006a; Arampatzis et al. 2007a; Kongsgaard et al. 2007; Seynnes et al. 2009]). Muscle has been shown to adapt to high mechanical loads and stretch (Tabary et al. 1972; McDonagh and Davies 1984). Like its in-series muscular counterpart, tendon appears to respond and adapt at varying magnitudes to the stimulus provided by mechanical loading and stretch (or strain) (Yang et al. 2004; Kubo et al. 2006a; Arampatzis et al. 2007a; Kongsgaard et al. 2007). For example, Arampatzis et al. (2007a) demonstrated that when training under cyclic low-magnitude tendon strain (2.97%) compared to high-magnitude tendon strain (4.72%) with equal volume and loading frequency, tendon–aponeurosis stiffness and Achilles tendon modulus increased significantly only following training under high-magnitude tendon strain. In parallel, Kubo et al. (2006a) trained the quadriceps and patella tendon MTC isometrically for 12 weeks at either a longer (LT) or shorter (ST) muscle length. Stiffness of the tendon–aponeurosis increased significantly (47%) following training in LT whereas ST did not (5%). Furthermore, the internal forces estimated from their data suggested that, based on the differences in the patella moment arm, although both groups trained at external torques of 70% maximal voluntary contraction (MVC), the LT group in fact experienced internal forces of 2090N compared to 908N in ST (i.e., ∼2.3 times greater). The above evidence suggests that the mechanics of the resistance exercise protocol has profound effects on the changes to tendon mechanical and material properties.

During dynamic resistance training, joint angle will change as bone rotates about its joint axis. A change in joint angle will also alter the length of the muscle–tendon unit. For example, the MTC length of the three monoarticular vasti (medialis, lateralis, and intermedius) of the quadriceps has been shown to increase by ∼15% during movement from full extension (0^o^) to 100^o^ of knee flexion in cadaveric specimens (Visser et al. 1990). This pattern has also been demonstrated in vivo, on both the gastrocnemius and tibialis anterior (Herbert et al. 2002). The authors found that passive movement of the ankle joint from full plantarflexion to dorsiflexion induced considerable changes in muscle–tendon unit lengths, with 72% and 45% of the total change in MTC length being accounted for by the tendons of the gastrocnemius and tibialis anterior, respectively. This demonstrates that a change in joint angle also alters the passive tension and therefore the strain experienced by the in-series tendon. In addition, altering joint angle during resistance training has been shown to alter the joint angle-specific expression of strength, and the range of joint angles to which significant increases in strength are attained (Thepaut-Mathieu et al. 1988; Kitai and Sale 1989; Kubo et al. 2006a). This has been demonstrated using isometric training; however, no studies provide insight into such relationships following a predominantly dynamic protocol.

At the other end of tendon loading spectrum, is chronic unloading such as seen in disuse where tendon has been shown to alter its mechanical properties in an almost inverse manner to those following training. De Boer et al. (2007) showed that after 14 and 23 days of unilateral lower limb suspension, patella tendon stiffness decreased by ∼10% and ∼30%, respectively, concluding that a time course exists in tendon adaptation to unloading. Alterations to the mechanical properties of tendon following bed-rest are in agreement with these findings (Kubo et al. 2004; Reeves et al. 2005). Similarly following a period of 3 months of isometric knee extensor training, (Kubo et al. 2010) reported a quasi-linear decrease in tendon stiffness measured at 1, 2, and 3 months posttraining, and that stiffness had returned to baseline values following 2 months of detraining. Evidently, a distinct difference exists between unloading and detraining, with detraining allowing habitual loading of the MTC through normal activity. Periods of detraining (such as through illness, injury and tapering) would often fall within a shorter time period of ≤4 weeks (i.e., less than the time-frame described by Kubo et al. 2010), and not necessarily result in bedrest/immobilization and therefore also allow for habitual loading. For example, this would allow for optimization of MTC function during a period of tapering in competitive performance, or to provide information as to the impact of a period of reduced activity on tendon properties where practitioners/clinicians could then gauge where to resume training or manipulate rehabilitation volume/intensity. However, no previous study has investigated if there is a distinct variation in tendon adaptation during the early and latter stages of a relatively shorter detraining period.

Alongside the in vivo effects of loading/unloading, there are many hormones, such as transforming growth factor beta (TGF-β1), that are associated with an exercise-induced response to mechanical loading of muscle and tendon. In patella tendon fibroblasts, TGF-β1 has been shown to increase proliferation of fibroblast number and to mediate increases in collagen Type I production following cyclical stretch (Yang et al. 2004). Furthermore from (Yang et al. 2004), TGF-β1 expression increased in a stretch-magnitude-dependent manner, with stretching of 4% and 8% resulting in greater levels of TGF-β1 mRNA compared to controls (i.e., no stretch). Similarly, in rodent models, TGF-β1 mRNA increased 27% following strain injury (induced from stretching) in the gastrocnemius muscle (Smith et al. 2007). Additionally, TGF-β1 mRNA increased significantly in muscle (gastrocnemius) and tendon (Achilles) following 4 days of resistance training with eccentric, isometric or concentric contraction types (Heinemeier et al. 2007). The picture however in humans is less well established. Heinemeier et al. (2013) took patella tendon and vastus lateralis biopsies following 1 h of kicking exercise at 67% workload max, showed TGF-β1 mRNA levels did not increase in tendon but TGF-β1/β2/βRII increased significantly in muscle. This is in agreement with another study that reported a significant increase in TGF-β1 mRNA in young and older men following downhill interval running in the vastus lateralis (Hamada et al. 2005). In contrast, previous work from Heinemeier et al. (2003) found significant increases in both systemic (plasma) and local (dialysate samples from peritendon tissue) of TGF-β1 levels immediately and 3 h post treadmill running, respectively (60 min, 3% incline, and 1 km/h). There is also evidence to suggest that chronic training can influence circulating levels of TGF-β1. In Type II diabetes patients who took part in 8 weeks of combined resistance training and aerobic training, TGF-β1 serum levels increased by 50.4% compared to baseline (Touvra et al. 2011). On the basis of these studies, one may expect that if there is a change in the dimensions of tendon (i.e., increased cross-sectional area [CSA] through stretch-induced collagen synthesis), that these could possibly be mediated, and indeed reflected, by changes in serum levels of TGF-β1. Indeed, it is clear from the above, that the chronic impact of exercise on circulating TGF-β1 in a young, healthy population is yet to be established.

The principal aim of this investigation was to describe the adaptations of the MTC to 8 weeks of a predominantly dynamic resistance exercise program, where the MTC would be performing excursions at three different average positions. In order to vary the magnitude of strain, two levels of excursions (0–50^o^ or 40–90^o^ knee flexion, 0^o^ = full extension) were to place the MTC in either a relatively shortened position (MTCS; 0–50^o^) or lengthened position (MTCL; 40–90^o^). Tendon forces (and work) during exercise were matched between these two groups to remove any impact of work differences. The third excursion range (which is normally encountered in a training program) was a combination of the entire first two ranges (MTCX; 0–90^o^ knee flexion) and this entailed working with higher internal tendon forces. It should be noted here that in terms of absolute external load, the tendon forces in MTCX were lower loads than seen in the MTCS condition – see explanation further on in the manuscript. The second aim of this study was to establish a time course of the quantitative/qualitative changes in MTC properties following a subsequent short period of detraining.

It was our hypothesis that the tendon would undergo a chronic stiffening response due to the resistance training protocol, the magnitude of which would present a step-wise increment (i.e., CON<MTCS<MTCL<MTCX) reflecting the combination of both force and strain placed on the MTC in each group. It was also hypothesized that increased strain during training would also be associated with a wider range of joint angles at which muscle forces would be increased chronically. Our third hypothesis was that the TGF-β1 levels would mirror the differences in strain magnitude between the groups. Our fourth and final hypothesis was that tendon does not alter its mechanical properties in a linear fashion during a relatively shorter period of detraining.

## Materials and Methods

### Subjects

Forty-two volunteers (physical characteristics Table 1) gave written informed consent to participate in this study. All procedures and experimental protocols were approved by the Ethics Committee at Manchester Metropolitan University. Exclusion criteria for participation in the study were the presence of any known musculoskeletal, neurological, inflammatory or metabolic disorders or injury. Participants were physically active, involved in recreational activities such as team sports, and had either never taken part in lower limb resistance training or not within the previous 12 months. Team sports included rugby union and league, soccer, hockey, and netball. Where several participants had the same sporting background, they were split up evenly and randomly allocated to a training group. For example, the six rugby union players that took part in the study were separated, with two randomly allocated to one of the three training groups. All participants took part in up to 5 h maximum of nonresistance based activity per week. Six male participants and four female participants (*n* = 10) were randomly assigned to the shorter MTC (MTCS) group, five male participants and six female participants were assigned to the longer MTC (MTCL) group (*n* = 11) and seven male participants and four female participants were (*n* = 11) assigned to the MTCX group. Ten participants (six men and four women) were assigned to the nontraining, habitual activity control group (CON).

**Table 1:** Participants' physical characteristics

Group	Height (m)	Mass (kg)	Age (years)
MTCS	1.76 ± 0.15	75.7 ± 13.2	19 ± 2.2
MTCX	1.71 ± 0.11	73.8 ± 14.9	19 ± 2.6
MTCL	1.75 ± 0.14	74.9 ± 14.7	21 ± 3.4
CON	1.76 ± 0.09	77.9 ± 13.1	23 ± 2.4

### Study design

The study design was convenience sampling, with random allocation to one of four groups. Following familiarization with testing procedures at least 1 week prior to baseline testing, participants and controls were tested for muscle–tendon properties at baseline (week 0), after 8 weeks resistance training (week 8), once again following 2 weeks of detraining (week 10) and finally after a further 2 weeks detraining (week 12), with all measurements recorded on the right leg. Testing was completed within 2 h of the time-of-day at week 0 to minimize any impact of diurnal variability in muscle function/tendon function (Martin et al. 1999; Pearson and Onambele 2005).

### Resistance training program

**Figure 1:**
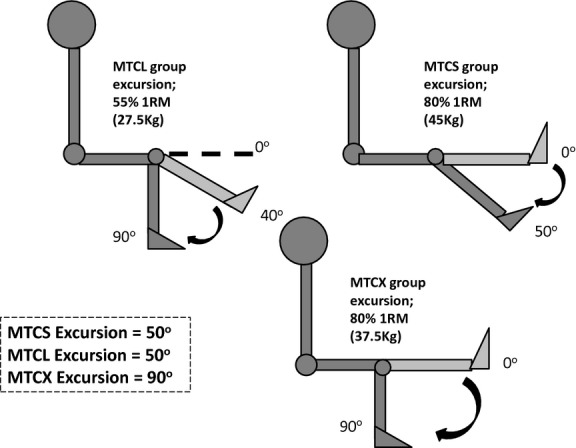
Diagram showing the ranges-of-motion, percentage (%) one repetition maximum, and a typical absolute load (kg) used in each of the three training groups MTCS (trained at shorter muscle–tendon complex length), MTCL (trained at longer muscle–tendon complex length), and MTCX (covered an entire range of muscle–tendon complex lengths i.e., shorter through longer) during the leg extension exercise. Note the difference in external load used to match internal tendon forces between MTCS and MTCL.

Resistance training was performed three times per week by the MTCS, MTCL, and MTCX training groups on Tuesdays, Thursdays, and Saturdays for 8 weeks, using a combination of free, machine (Technogym, Berkshire, U.K.) and body weights to complete the exercises. Dynamic exercises included the barbell back squat, Bulgarian split squat, leg press, leg extension, and dumbbell lunges. In addition, a static loading exercise was also included (Sampson chair). Exercises were performed on both legs, and were chosen as they are frequently used in the strength and conditioning/clinical rehabilitation environments for conditioning of the knee extensor complex and/or lower limbs. All exercise sessions were supervised by a member of the research team. A generalized warm-up was completed at 70–75% age-predicted maximum heart rate on a treadmill for 5 min (Thompson et al. 2009), after which a goniometer was attached to the center of rotation of the knee. The training group excursions are shown in Figure 1. Briefly, the MTCS group performed all exercises from 0^o^ (full extension) to 50^o^ knee flexion, MTCL from 40^o^ to 90^o^ knee flexion and MTCX group from 0^o^ to 90 knee flexion. The MTCL group received the weight at 40^o^ knee flexion and unloaded on the final rep at 40^o^ knee flexion to make sure they did not perform extra work at the beginning or end of each set. All exercises involved eccentric and concentric contractions with an isometric contraction held for 2 sec at the end range-of-motion joint angle, before returning to the desired starting joint angle. Joint angles were confirmed by a training partner from the goniometer scale (each joint angle to be achieved was clearly marked) and exercise time frame rhythmically controlled using a metronome. The subjects completed two familiarization sessions at 70% of 1 repetition maximum (1RM) prior to commencing the resistance training program a week later.

During the training program, exercises were performed at 80% of 1RM in MTCS and MTCX groups, whereas MTCL performed exercise at 55% of 1RM (see Muscle force modeling). 1RMs were determined as the maximal amount of weight that the participant could lift covering the excursion of their designated training group (e.g., a MTCS participant performed a 1RM between 0^o^ and 50^o^; MTCL between 40^o^ and 90^o^) 1RMs were measured every 2 weeks (first determined at week 0) and loading weight adjusted accordingly. Volume (in terms of repetitions and sets) was identical for each training group, with each training session consisting of four exercises and performing three sets of 10 repetitions per exercise for the first 4 weeks, and four sets of eight repetitions per exercise thereafter. In order to not exceed a training volume attainable by our study participants, four of the six exercises (two squat exercises, one machine-based exercise and a Sampson chair) were performed in each session.

### Muscle force modeling

Due to the changing moment arm length of the patella tendon at discrete knee joint angles, differences in muscle force produced between the groups had to be accounted for. Thus quadriceps forces at the patella tendon were calculated as follows:


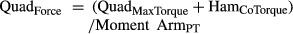
(1)

where


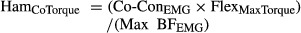
(2)

Where Quad_MaxTorque_ is maximum knee extensor torque, Ham_CoTorque_ is Hamstring cocontraction torque (i.e., the activity of this muscle during Quad_MaxTorque_), Moment Arm_PT_ is the moment arm of the patellar tendon (PT) (see Patella tendon moment arm measurement), Co-Con_EMG_ is the electromyographic (EMG) activity recorded from the biceps femoris acting as an antagonist muscle that is, cocontracting, and Max BF_EMG_ is the maximum EMG of the biceps femoris when in agonist mode. Flex_MaxTorque_ is maximum flexion torque. Based on tendon forces estimated from the 1RM data at the end range-of-motion from the MTCS (50^o^) and MTCX (90^o^) training groups (i.e., where a short isometric hold would take place), tendon forces produced at 90^o^ were on average ∼32% greater than those produced at 50^o^.

Thus, in order to quantify the training load to apply, the weight of the external resistance (converted to the equivalent torque) is added to the left hand side of equation (1) above. Hence, it was calculated that while MTCS and MTCX would exercise at a high intensity of 80% 1RM (for a more detailed description see Resistance training program section), MTCL would train at a lower intensity of 55% 1RM to equate the absolute load seen by the tendon in the MTCS/L groups. In order to accurately assess internal muscle forces produced, the change in resistance moment arms of the cam pulley machine used during leg extensions were also measured. Based on the training load for the leg extension exercise, the resistance machine load component yielded on average a 7% increase in external torque produced in the MTCS group compared to MTCX, and was therefore accounted for, for MTCL (MTCS; 137 Nm vs. MTCX; 128 Nm).

### Strength and torque-angle measurement

Maximal isometric knee extension torque was measured with the knee at a range of angles that is 30°, 50°, 60^o^, 65°, 70°, 75°, and 90° (full knee extension = 0°) on the right leg of all participants. The order of testing by knee angle was randomized so as to minimize any systematic fatigue effect. After a series of warm up trials consisting of 10 isokinetic contractions at 60°·s^−1^ angular velocity at 50–75% of self- perceived maximal effort, participants were instructed to rapidly exert maximal isometric force against the dynamometer (Cybex; Phoenix Healthcare, Birmingham, U.K.) lever arm. Participants were given both verbal and visual encouragement/feedback throughout their effort. Joint torque data were displayed on the screen of a MacBook Air computer (Apple Computer, Cupertino, CA), which was interfaced with an A/D system (Acknowledge, Biopac Systems, Santa Barbara, CA) with a sampling frequency of 200 Hz. Isometric contractions were held for ∼2 sec at the plateau with a 60 sec-rest period between contractions. Peak torque was expressed as the average of data points over a 200 msec period at the plateau phase (i.e., 100 msec either side of the instantaneous peak torque). The single greatest value for peak torque of three extensions was used as the measure of strength for each participant.

### Patella tendon moment arm measurement

**Figure 2:**
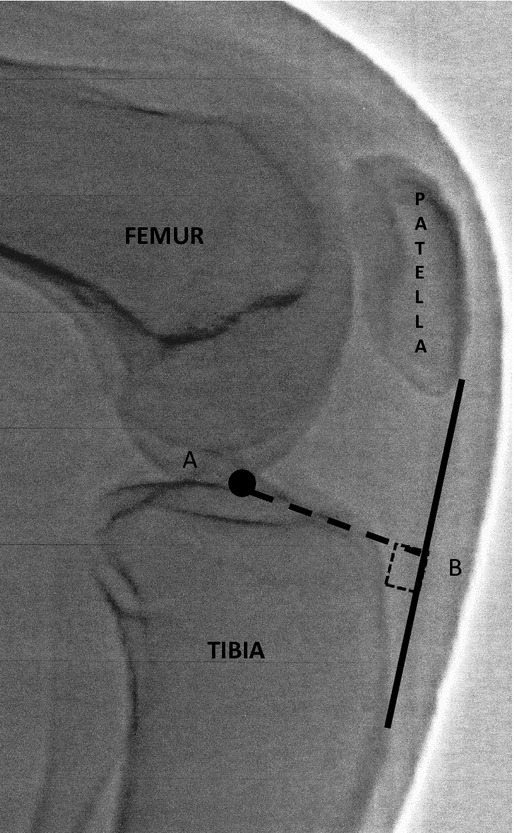
Typical sagittal plane image taken by Dual Energy X-ray Absorptiometry (DEXA) with the knee at 90^o^ flexion for measurement of patella tendon moment arm. The moment arm is the line A–B (i.e., the perpendicular distance from the joint center of rotation to the line of force application).

The patella moment arm was estimated from sagittal scans of the right leg of each participant using a DEXA (Dual Energy X-Ray Absorptiometry) scan (Hologic QDR, Vertec, Reading, U.K.) in single-energy mode, with the knee placed at 90^o^ of knee flexion. The patella tendon moment arm was defined as the perpendicular distance between the tibiofemoral contact point and the mid-portion of the patella tendon (Fig. 2). DEXA imaging has been used to estimate moment arm previously in other anatomical sites, with good reliability (Wang et al. 2009).

### Tendon properties

Tendon elongation was determined using ultrasound imaging (7.5-MHz, 40-mm linear array, B-mode ultrasound probe [AU5; Esaote Biomedica, Genoa, Italy]) set to a depth resolution of 55.3 mm, and placed over the apex of the patella in the sagittal plane, with the knee fixed at 90^0^ flexion. Forces were determined by adding the measured external torque (applied to the dynamometer) to the cocontraction torque (calculated from the antagonistic muscles EMG activity). Before testing proper, five preconditioning trials were carried out to ensure reproducibility. Although the current protocol only allowed the quantification of proximal tendon displacement, this did not detract from the validity of the data gathered as (1) this “partial visualization” technique has been detailed and validated previously (Reeves et al. 2003; Pearson and Onambele 2005), and (2) tendon displacement is a relative parameter, (3) this study used a within-subject design. Briefly, an echo-absorptive marker was placed at 25% proximally of tendon length between the probe and the skin to act as a fixed reference from which relative measures of displacement could be made. Following each completed contraction (6-sec graded isometric ramp with a constant low rate of force development), the distance between the original position of the tissue under the skin, relative to the new position of the tissue was recorded. Ultrasound images were recorded in real-time and captured onto a personal computer at 25 Hz. The ultrasound output was synchronized using a square wave signal generator (homemade) to allow temporal alignment with the torque and EMG data. Tendon displacement was determined at intervals of 10% of the maximal force (from 10% to 100%) using image J (National Institutes of Health, Bethesda, MD). Three efforts were recorded, analyzed, and the average reported as the profile of tendon force versus strain for the participant.

**Figure 3:**
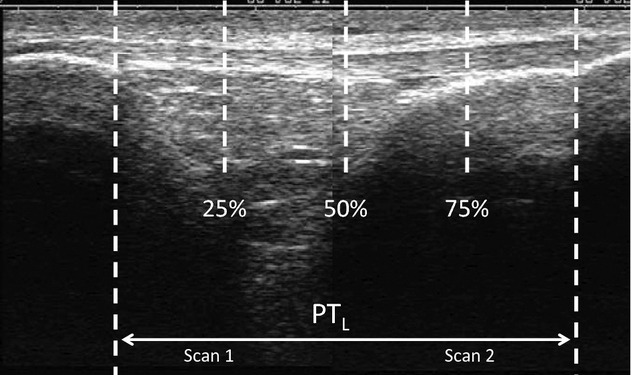
Sagittal plane ultrasound image (composed of two scans) of the patella tendon denoting measurement locations of tendon cross-sectional areas at 25%, 50%, and 75% of total patella tendon length (knee at 90^o^ flexion).

Tendon forces were then calculated as: Measured Torque (corrected for antagonist coactivation)/Patella Moment Arm. The plotted force–elongation relationship was fitted with a second-order polynomial function, forced through zero. Tendon stiffness (K in N mm^−1^) was calculated from the slope of the tangents at 10% force intervals. To allow between subjects comparisons at a set force, tendon stiffness was calculated at a standardized force level (2000N), which corresponded to just under the maximum baseline force value of the weakest person. Patellar tendon resting length (PTL) and cross-sectional area (PTCSA) were assessed with the knee joint at 90^o^ of flexion. PTL was measured from the inferior pole of the patellar to the superior aspect of the tibial tuberosity as determined from sagittal-plane ultrasound images (see Fig. 3). PTCSA was determined from the mean of transverse plane ultrasound images taken at 25%, 50%, and 75% PTL. Young's modulus was computer as instantaneous stiffness multiplied by the ratio of resting PL/PTCSA.

### Electromyography

EMG activity of a representative muscle in the knee flexors (long head of the biceps femoris) was measured to correct for coactivation during isometric knee extension. The skin was prepared by shaving, abrading, and cleaning with an alcohol-based solution to minimize resistance (i.e., reduce skin impedance to values below 5 kΩ). A pair of self-adhesive Ag–AgCl electrodes 15 mm in diameter (Medicotest, Rugmarken, Denmark) was then placed in a bipolar configuration with a constant interelectrode distance of 20 mm, at 50% of total femur length. These electrodes were placed in the midsagittal plane of the muscle, and the reference electrode was placed on the lateral tibial condyle. The raw EMG signal was preamplified (×2000) and band pass-filtered between 500 Hz and 10 Hz, sampled at a rate of 2000 Hz by the same system that handled the torque data (Biopac Systems). Signals were displayed in real-time on the same graph so that all data would be synchronized (MacBook Air, Apple, CA). The reported EMG activity in this study corresponds to the root mean square (RMS) after correcting for baseline values.

The RMS EMG activity corresponding to the peak torque period was analyzed and averaged for 1-sec peak torque duration. As mentioned earlier, the EMG of the long head of the biceps femoris muscle was measured to ascertain the level of antagonist muscle cocontraction during the required isometric knee–extension performances. The biceps femoris torque during a knee–flexion contraction was calculated in a manner similar to the method described by Reeves et al. (2003), where the biceps femoris EMG activity during knee extension is divided by the biceps femoris peak flexor EMG at 90^o^ knee flexion; the maximal flexor torque is then multiplied by this value to determine cocontraction torque. The cocontraction torque values are used to correct the voluntary knee–extension torques (and hence the forces during the ramped contractions) using the following formula:


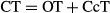
(3)

where CT represents corrected knee–extensor torque, OT is the observed knee–extensor torque, and CcT is the calculated hamstrings torque during knee extension (i.e., antagonist cocontraction torque).

### MTC dimensions as a function of joint angle

In order to quantify and contrast the strain in the training groups, MTC length and PTCSA (25%, 50%, 75% of PTL) were measured at rest at the varying knee angles used in the study (0^o^ – full extension, 40^o^, 50^o^, and 90^o^) in a subsample of our study population (*n* = 7). Using B-mode ultrasonography as above, this was measured as a line passing directly from the proximal musculo-tendinous junction of the vastus lateralis muscle, to the superior aspect of the tibial tuberosity. The results are displayed in Table 2.

**Table 2:** Muscle–tendon complex lengths at various knee joint angles

Knee joint angle (^o^ knee flexion)	MTC length (cm)	PTCSA (mm^2^)			
0^o^ (Full extension)	36.5 ± 2.9	25	89 ± 23
		50	94 ± 14
		75	90 ± 17
40^o^	38.9 ± 2.4^1^	25	84 ± 9
		50	93 ± 10
		75	101 ± 9
50^o^	39.7 ± 2.4^1^	25	86 ± 10
		50	79 ± 12
		75	98 ± 5
90^o^	42.0 ± 2.7^2^	25	83 ± 11
		50	87 ± 16
		75	86 ± 11

Values are mean ± SD.

1 Significantly different from full extension (*P* < 0.05).

2 Significantly different from full extension (*P* < 0.0001) *n* = 6.

#### Circulating TGF-β1 levels

At each of the designated testing intervals (i.e., baseline, weeks 8, 10, and 12) and following an overnight fasting period (∼10 h for all participants), participants reported to the laboratory. A 21-gauge 1-inch ultrathin wall needle (Terumo Medical Corporation, Somerset, NJ) was inserted into the antecubital vein of the forearm. Using a vacutainer assembly and serum separator tubes (Monovette, Sarstedt, Numbrecht, Germany), 5 mL blood samples were collected. After being kept on an ice bed for up to 2 h, the sample was then centrifuged at 4°C for 10 min at 6193 *g*, with the supernatant being removed and stored in eppendorfs at −20°C for later analysis. The standard enzyme-linked immunosorbent assay (ELISA) technique was used to quantify TGF-β1 (R&D Systems Europe, Abingdon, U.K.; sensitivity 4.61 pg/mL; serum/plasma intraassay variability of 2.5% CV, manufacturer's data, 3.3% CV this study CV) levels. The optical density of the wells was analyzed using a microplate reader (EL808; Biotek, Bedfordshire, U.K.) set to 450 nm.

### Statistics

Data were analyzed using SPSS version 19.0.0 (Armonk, NY). Following parametricity checks, a mixed design repeated measures 4 × 4 analysis of variance (ANOVA) was used. The “within factor” was the phase of training (i.e., week 0, 8, 10, and 12) and the “between factor” was training group (i.e., MTCS, MTCL, MTCX, and CON). Post hoc contrast analyses with Bonferroni corrections were used to compare data to baseline (within factor) and to control group (between factors). All data are presented as mean (±SD). Statistical significance was set with α ≤ 0.05.

## Results

### Strength and torque-angle relationship

**Figure 4:**
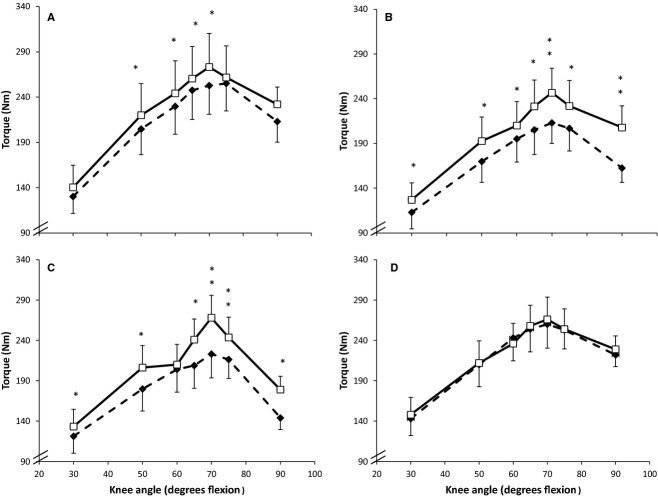
Changes in torque levels at various joint angles at baseline (dashed line) and at week 8 (solid line) in (A) MTCS, (B) MTCX, (C) MTCL, and (D) CON. *Significantly different relative to baseline (*P* < 0.05). **Significantly different relative to baseline (*P* < 0.01).

There was a significant main effect of training on all three training groups (*P* = 0.013), with each group displaying varying magnitudes of torque increment at each angle. MTCS achieved a significant (*P* < 0.05) relative increase in torque at four angles (mean Δ6 ± 4%; 50^o^, 60^o^, 65^o^, 70^o^). MTCX torque increased at six angles (mean Δ16 ± 5%; 50^o^, 65^o^, 75^o^ *P* < 0.05; 30^o^, 70^o^, 90^o^ *P* < 0.01), whereas MTCL recorded a significant relative increase in torque at all seven angles (mean Δ21 ± 8%; 30^o^, 50^o^, 60^o^, 65^o^, 90^o^ *P* < 0.05; 70^o^, 75^o^ *P* < 0.01). Following training at week 8, maximal torque increases were 7 ± 3%, 15 ± 2% and 26 ± 6% for MTCS, MTCX, and MTCL, respectively, with MTCX and MTCL being significantly different to MTCS (*P* < 0.05). Following detraining at week 12, MTCS did not differ from baseline (*P* > 0.05), whereas MTCX (11 ± 4%) and MTCL (19 ± 7%) remained elevated compared to baseline (*P* < 0.05). There was no change in the angle of peak torque production from pretraining to posttraining in MTCL and MTCX groups (70^o^). Interestingly, angle of peak torque production shifted from 75^o^ pretraining to 70^o^ posttraining in MTCS. There was no significant change in strength at any angle, or angle of peak torque in the control group (*P* > 0.05). Strength and torque angle data are presented in Figure 4.

### Tendon dimensions and antagonist cocontraction

Changes in tendon dimensions and cocontraction of the biceps femoris are shown in Table 3. There was no significant change in tendon length or CSA at 25%, 50% or 75% of PT length following training and detraining in any training or control group (*P* > 0.05). Additionally, no change in maximal antagonist activation or cocontraction during 100% knee extension MVC were detected at any stage of the study (*P* > 0.05). Changes in tendon dimensions and cocontraction of the biceps femoris are shown in Table 3.

**Table 3:** Presentation of data on tendon dimensions and properties, and antagonist cocontraction

Variable	Baseline (wk 0)				Posttraining (wk 8)				Detraining 1 (wk 10)				Detraining 2 (wk 12)			
			
	MTCS	MTCL	MTCX	CON	MTCS	MTCL	MTCX	CON	MTCS	MTCL	MTCX	CON	MTCS	MTCL	MTCX	CON
PT length (mm)	52.3 ± 4.7	53.1 ± 5.3	50.5 ± 5.1	49.8 ± 4.4	52.0 ± 4.4	53.9 ± 5.4	50.8 ± 4.9	49.2 ± 5.1	52.1 ± 4.5	54.1 ± 5.4	51.3 ± 5.1	49.0 ± 4.9	52.0 ± 4.4	53.8 ± 5.0	50.9 ± 4.7	50.5 ± 5.6
PTCSA (mm^2^)																
25%	67 ± 11	69 ± 14	71 ± 11	70 ± 8	69 ± 12	76 ± 18	74 ± 15	71 ± 8	67 ± 14	74 ± 18	75 ± 12	70 ± 8	68 ± 8	73 ± 16	70 ± 14	71 ± 10
50%	70 ± 9	78 ± 13	69 ± 13	73 ± 10	74 ± 15	83 ± 13	75 ± 16	72 ± 12	74 ± 10	82 ± 13	73 ± 11	71 ± 13	71 ± 10	82 ± 12	73 ± 15	72 ± 12
75%	74 ± 18	69 ± 15	73 ± 15	71 ± 12	77 ± 15	71 ± 25	78 ± 17	71 ± 12	75 ± 17	77 ± 13	77 ± 16	72 ± 11	73 ± 16	77 ± 13	75 ± 15	71 ± 15
Max flexion RMS-EMG (V)	0.55 ± 0.30	0.50 ± 0.23	0.69 ± 0.42	0.72 ± 0.35	0.48 ± 0.24	0.47 ± 0.23	0.57 ± 0.18	0.70 ± 0.38	0.47 ± 0.37	0.35 ± 0.25	0.52 ± 0.30	0.70 ± 0.47	0.48 ± 0.31	0.43 ± 0.23	0.67 ± 0.33	0.73 ± 0.38
Cocontraction RMS-EMG (mV)	0.046 ± 0.024	0.058 ± 0.050	0.044 ± 0.015	0.052 ± 0.019	0.050 ± 0.037	0.045 ± 0.040	0.046 ± 0.030	0.047 ± 0.018	0.047 ± 0.028	0.043 ± 0.026	0.054 ± 0.027	0.049 ± 0.020	0.045 ± 0.032	0.046 ± 0.035	0.054 ± 0.032	0.052 ± 0.014
PT stiffness (N mm^−1^)	916 ± 441	837 ± 379	765 ± 242	997 ± 258	1221 ± 594^1^	1124 ± 471^1^	1167 ± 353^1^	1014 ± 218	1115 ± 530^1^	1206 ± 503^1^	1070 ± 346^1^	1024 ± 222	1024 ± 439	1043 ± 428^1^	989 ± 344^1^	1011 ± 231
Δ Relative stiffness (%)	–	–	–	–	35 ± 6^1^	43 ± 11^1^,^2^	50 ± 18^1^,^2^	2 ± 3	23 ± 12^1^	54 ± 16^1^,^2^	43 ± 17^1^,^2^	3 ± 3	14 ± 8	35 ± 14^1^,^2^	32 ± 11^1^,^2^	2 ± 3
Δ Relative stiffness 2kN (%)	–	–	–	–	38 ± 19^1^	43 ± 14^1^	51 ± 30^1^	1 ± 3	17 ± 18^1^	51 ± 19^1^,^2^	31 ± 17^1^,^2^	2 ± 3	14 ± 14	33 ± 16^1^,^2^	38 ± 18^1^,^2^	1 ± 3
Young's modulus (GPa)	0.83 ± 0.09	0.74 ± 0.09	0.78 ± 0.10	0.89 ± 0.14	1.10 ± 0.12	0.99 ± 0.11	1.15 ± 0.11	0.92 ± 0.11	1.02 ± 0.10^1^	1.03 ± 0.11	1.11 ± 0.12	0.93 ± 0.14	0.91 ± 0.05	0.91 ± 0.13	1.02 ± 0.14	0.95 ± 0.16
Δ Relative Young's modulus (%)	–	–	–	–	36 ± 7^1^	33 ± 5^1^	48 ± 6^1^,^2^	3 ± 3	23 ± 7^1^	40 ± 5^1^,^2^	42 ± 12^1^,^2^	3 ± 5	13 ± 2^1^	24 ± 13^1^,^2^	31 ± 13^1^,^2^	6 ± 6

Values are mean ± SD. PT, patella tendon.

1 Significantly different from baseline (*P* < 0.05).

2 Significantly different to MTCS (*P* < 0.05).

### Patella tendon properties

**Figure 5:**
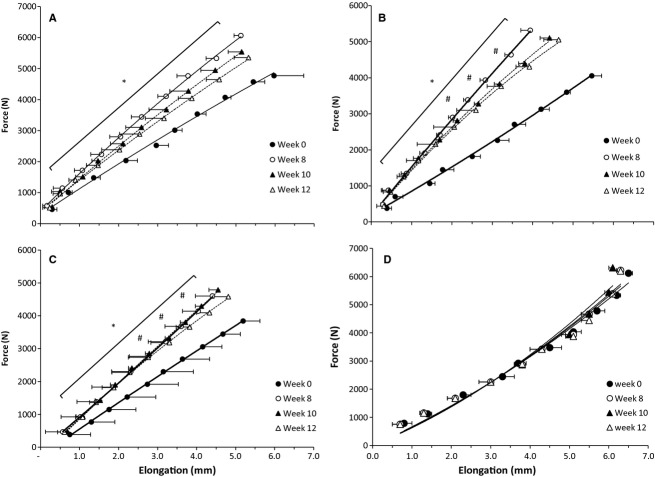
Changes in patella tendon force–elongation relationship from 10% to 100% MVC at baseline, posttraining (week 8) and detraining (week 10 and week 12) in (A) MTCS, (B) MTCX, and (C) MTCL training groups, and (D) CON. *Significantly different to baseline. #Significantly different to MTCS.

In MTCS, there was a significant effect of training (*P* < 0.001) at week 8 on tendon elongation at force levels of 40–100% MVC, while MTCL and MTCX displayed a main training effect (*P* < 0.001) at force levels of 30–100% MVC at week 8. Also at week 8, there was a group effect with MTCL and MTCX having significantly less tendon elongation at force levels of 70% (*P* = 0.017), 80% (*P* = 0.007), and 90% (*P* = 0.005) MVC, with a trend for a group effect at 100% MVC (*P* = 0.07). Following detraining, elongation values had returned to baseline at week 12 in MTCS; whereas it remains significantly above baseline (*P* < 0.001) at week 12 in both MTCL and MTCX groups. There was no significant change in tendon force–elongation throughout the study in the CON group (*P* > 0.05). Figure 5 shows tendon force–elongation curves, and changes to tendon stiffness (K) at each measurement period are in Table 3.

Average Patella tendon stiffness across the entire range of normalized forces increased posttraining in all training groups (*P* < 0.001) compared to baseline, with relative increases in K significantly greater in both MTCL (43 ± 11%) and MTCX (50 ± 18%) groups compared to MTCS (35 ± 6%; *P* < 0.05). Interestingly following 2 weeks of detraining, MTCL group K increased further by 11% (i.e., to 54 ± 16%), whereas K decreased in the other training groups (−7 ± 4% MTCX; −12 ± 6% MTCS). There was, however, no difference between MTCL and MTCX (*P* > 0.05) at this stage. Variable patterns of tendon stiffness change persisted throughout the detraining period with MTCL and MTCX groups remaining above baseline (*P* < 0.05) following detraining at weeks 10 and 12, whereas MTCS had returned to baseline values by week 12. CON group stiffness did not change significantly at any stage of the study (*P* > 0.05).

Relative changes in patella tendon stiffness at a standardized tendon force (2000N), followed a similar trend to those at all force levels. At week 8, there was a main effect of training observed (*P* < 0.001), though no significant effect of group evident (*P* > 0.05). Main effects of training phase (*P* < 0.001) and group (*P* < 0.05) were shown at week 10 with MTCS (17 ± 18%) once again displaying reduced adaptations compared to both MTCL (51 ± 19%) and MTCX (31 ± 17%; *P* < 0.05). Following the conclusion of the detraining period at week 12, relative changes in stiffness values at 2000N in MTCL (33 ± 16%) and MTCX (38 ± 18%) remained significantly enhanced compared to baseline (*P* < 0.05), although MTCS tendon stiffness at that force level did not (14 ± 14%; *P* > 0.05). Neither stiffness at 2000N, nor the force–elongation relationship significantly changed (*P* > 0.05) at any stage of the 12-week period in the CON group (see Table 3).

Tendon Young's modulus increased significantly in all training groups following resistance training (*P* < 0.001), with MTCL and MTCX displaying a greater relative increase (*P* < 0.05) compared to MTCS at this stage. The MTCL and MTCX groups also had significantly greater Young's modulus compared to MTCS (*P* < 0.05) following detraining at weeks 10 and 12. Interestingly, all training groups remained above baseline values at week 12 (*P* < 0.05). The CON group's Young modulus did not change significantly (*P* > 0.05) at any stage of the study (see Table 3).

### Circulating growth factor levels

After 8 weeks of resistance training, circulating levels of TGF-β1 did not change significantly in MTCS (4976 ± 1279 pg/mL to 4274 ± 1071 pg/mL) or MTCL (4572 ± 807 pg/mL to 4755 ± 752 pg/mL) training groups (*P* > 0.05), with no differences observed between groups (*P* > 0.05). Following 4 weeks of detraining TGF-β1 levels remained similar to those reported at baseline and at week 8 in both training groups (MTCS; 3726 ± 519 pg/mL, MTCL; 4439 ± 815 pg/mL).

## Discussion

This study aimed to describe the changes to the MTC under chronic differential strain. This was achieved through following 8 weeks of resistance training with the MTC in a relatively MTCS, MTCL or moving from a shortened position into a lengthened position (i.e., a range-of-motion normally encountered in resistance training; MTCX). In line with our primary hypothesis, the MTCL group showed increased PT stiffness following training compared to MTCS. Surprisingly, MTCL and MTCX (despite greater tendon stresses in MTCX compared with MTCL) groups displayed no difference in relative training-induced increments in tendon stiffness (*P* > 0.05). This therefore led us to also support our second hypothesis that increased strain (and not necessarily greater degree of motion) during loading would be associated with further enhanced tendon stiffening. In fact, our data suggest for the first time in human that in vivo, it is the degree of strain (but not the width of the range of motion or the absolute tendon stress), which is key to tendon adaptations. Circulating TGF-β1 levels did not change significantly in either MTCS or MTCL training groups compared to baseline, but on average tended to decrease over the course of training and detraining, from 4774 pg/mL at week 0 to 4082 pg/mL at week 12 (mean MTCS + MTCL). Therefore, we must reject our third hypothesis that systemic TGF-β1 levels would mirror the degree of strain in the tendon.

Following resistance training, there are concurrent adaptations to both the force transmitting connective tissue that is tendon, as well as the contractile unit (Kongsgaard et al. 2007; Seynnes et al. 2009). Following prolonged periods of resistance training, it has been demonstrated that tendon (e.g., patella and Achilles) has the ability to adapt both morphologically (Arampatzis et al. 2007a; Kongsgaard et al. 2007; Seynnes et al. 2009) and in terms of intrinsic material properties (i.e., increased stiffness and/or Young's modulus [Reeves et al. 2003; Maganaris et al. 2004; Kubo et al. 2006a; Arampatzis et al. 2007a; Kongsgaard et al. 2007; Seynnes et al. 2009]). Previous research has suggested that the resistance training mechanics (i.e., forces transmitted through the tendon) appear to be major mechanical stimuli for enhancing tendon adaptations (Kubo et al. 2006a; Arampatzis et al. 2007a; Kongsgaard et al. 2007). However, to our knowledge, no study has previously attempted to systematically differentiate between the impacts of load versus strain per se on the chronic adaptations to resistance training. In this study, to bring about the effects of strain, we positioned the MTC during dynamic training over two different ranges-of-motion. Evidence from cadaveric specimens (Visser et al. 1990) and from in vivo research (Herbert et al. 2002) has shown that the MTC is lengthened to varying levels depending on the joint angle achieved. We also reported the lengths of the MTC during the joint angles used in this study to illustrate that the MTC was significantly more lengthened at 90^o^ compared with 50^o^ of knee flexion, or with full-range of motion (Table 2). On the strength of this assumption, we had anticipated that with MTCS covering a range-of-motion of 0–50° and MTCL a range-of-motion of 40–90°, the latter would be experiencing greater MTC strain, on average, under loading and thus greater training stimulus. Our data support this hypothesis because tendon stiffness increment was significantly greater in this study in the MTCL group (43 ± 11%) compared to MTCS (35 ± 6%), (which also translates to significantly less elongation of the tendon at 30%, 70%, 80%, and 90% of MVC in MTCL following training).

In ligaments, cyclic stretch has been shown to increase expression of both Type I and Type III collagen, and also the expression of TGF-β1, which plays an important role in connective tissue remodeling (Kim et al. 2002). With regard to the adaptation of the MTC, TGF-β1 plays a pleiotropic role. Specifically with regard to the tissue of interest here, in patella tendon fibroblasts, TGF-β1 has been shown to increase proliferation of fibroblast number and at least in part mediate increases in collagen Type I (Abrahamsson and Lohmander 1996; Yang et al. 2004). Yang et al. (2004) investigated the effects of cyclical uniaxial stretching on tendon fibroblast numbers and collagen production. Fibroblasts were either stretched by 4%, 8% or not stretched (control) with both stretch frequency and duration kept constant. The results showed that tendon fibroblast proliferation only increase significantly following stretching at 8%, with Type I collagen level and TGF-β1 mRNA levels both increasing in a stretch–magnitude-dependent manner. In this study, serum TGF-β1 levels did not change significantly through the course of either the training or detraining phases, in MTCS or MTCL groups. The fact that TGF-β1 levels did not change compared to baseline and between groups, together with the lack of changes in tendon CSA (along the length of the patella tendon) in any training group, is supportive of the evidence of changes in the intrinsic properties of this unit rather than any tendon hypertrophic training-induced response being a stress or strain-mediated one.

From this evidence, cadaveric data, plus that presented in Table 2, we propose that the stimulus for the observed adaptations in addition to the forces produced by the knee extensors, was the stretch experienced by the MTC over 8 weeks of training in MTCL. This stretch led to an accumulation of cellular responses resulting in the differential tendon properties changes observed between the two groups. However, the signaling pathway for the variation in responses remains to be elucidated. Here, the muscular work performed between MTCS and MTCL was in fact matched (both groups covered an excursion of 50^o^ with forces at the tendon also equated), so that the loading histories of the tendon would be very similar, and therefore not result in group differences (Seynnes et al. 2009). Although MTC strain was not measured directly under contraction during training, previous work supports the idea that the training protocols gave rise to differential levels of strain in the MTC (Herbert et al. 2002). Indeed, Karamanidis and Arampatzis (2006) showed that passive elongation of the triceps surae and quadriceps femoris MTCs are very similar (∼7 and 6 mm, respectively). These authors also demonstrated that maximal strain is only slightly greater in the quadriceps femoris compared with the triceps surae (7.2% vs. 6.2%) in young runners, with the same pattern also evident in controls. What is more, Fukunaga et al. (1997) showed that during knee flexion (both at rest and under contraction) at longer muscle lengths, *vastus lateralis* (VL) fascicles shorten much less than at shorter lengths, thereby demonstrating that the tendon takes up the “slack” in the MTC and is therefore experiencing a degree of strain. In other words, the data from Herbert et al. (2002) along with the fascicle data from Fukunaga et al. (1997) even if these are on the triceps surae, provide a strong basis for suggesting that passive strain would be occurring in the quadriceps femoris MTC to different extents between the MTCS/L groups, with changes in joint angle in this study. In addition to the difference between MTCS and MTCL training-induced changes, there were no significant differences between stiffness and elongation measurements in the MTCL compared with the MTCX groups (43 ± 11% vs. 50 ± 18%). This was somewhat surprising, as the MTCX group would have had a more extensive loading history (in terms of muscular work done; 90^o^ excursion) and also experienced greater peak tendon forces due to exercising at 80% 1RM compared to MTCL who only exercised at 55% 1RM. The fact that the combined effects of higher contraction duration, wider contraction range-of-motion and higher absolute load resulted in a lesser stiffening of the tendon compared with that where the MTC was under constant lengthening (and comparatively lower load) would support the idea that the relative contribution of strain (i.e., stretch) to tendon adaptation may indeed be more substantial than previously considered.

The strain-dependent tendon stiffening has implications for the applied setting. For example, it has been shown that sprinters have stiffer tendons/tendon aponeurosis than endurance or nonactive counterparts (Arampatzis et al. 2007b). Interestingly, sprint performance between fast and slow sprinters is negatively correlated with the elongation of the vastus lateralis and its tendon (Stafilidis and Arampatzis 2007). These observations indicate that tendon properties affect performances during stretch-shortening cycle (SSC) exercises. Although stiffness would enhance transmission of forces to bone, the reduced elastic return during SSC exercises in fact has a negative impact. Indeed, Kubo et al. (2006b) found that although tendon stiffness increases following 12 weeks of isometric squat training, while performance of the squat jump increased, countermovement jump did not improve. Similarly, Kubo et al. (2007) found that after 12 weeks of: (1) weight training, tendon stiffness and squat jump performance increased whereas, after (2) plyometric training for a similar duration of time, there was no change in tendon stiffness but an improvement in squat jump, countermovement jump and drop jump performance.

In addition to the above findings, the current findings suggest that when altering external loads in the resistance training setting, the range-of-motion should also be closely monitored. Indeed our data support the idea that range-of-motion should be modulated, not only to (1) enhance the adaptations of the MTC and its ability to retain the training-induced biomechanical improvements but also (2) to allow the individual to be better able to recover from the “sticking point” of heavy-resistance exercise, and hence, able to produce a more complete and safe lift.

Notably also, there were no changes in patella tendon morphology following training or detraining in any of the training groups or control groups, which is in agreement with another study measuring PTCSA proximally, centrally, and distally by ultrasound (Reeves et al. 2003). Other studies have reported region-specific increases in PTCSA following prolonged resistance training (Kongsgaard et al. 2007; Seynnes et al. 2009). Kongsgaard et al. (2007) measured tendon CSA at proximal, mid and distal areas along the length of the PT by magnetic resonance imaging (MRI), with the knee placed at 90^o^ flexion which is similar to this study. Differences may have arisen due to a number of reasons. First, there may have been minute discrepancies at the exact intervals of where the measurement sites occurred. They measured PTCSA just distal to the patellar insertion (i.e., at ∼0% of tendon length), just proximal to the tibia insertion (i.e., at ∼100% of tendon length), and midway between those two points (i.e., at ∼50% of tendon length). In this study we measured tendon CSA at 25%, 50%, and 75% of total tendon length (apex of patella to tibial tuberosity). The research of Seynnes et al. (2009) using MRI shows that in fact along the length of the tendon, there are only five of 10 discrete areas in which there is evidence of region-specific tendon hypertrophy with chronic training stimulus. This highlights the degree to which small interstudy differences in methods may lead to contradicting findings. Indeed, Seynnes et al. (2009) report significant region-specific hypertrophy very close to the midregion of the tendon. It is noteworthy that the tendon areas that underwent significant training-induced hypertrophy in Seynnes et al. (2009), are in fact extremely close to the areas measured in this study, thereby highlighting that measurement site location may not be the only between studies discrepancy. Another confounding factor when comparing the studies is the gender of the participants involved. This study used an approximately equal number of male participants and female participants, whereas the two aforementioned studies reporting hypertrophy used men only. Considering that Miller et al. (2005) showed that following exercise, women do not have the same elevated levels of protein synthesis rate of tendon collagen; and that it has also been reported that patella tendon CSA is significantly greater in well-trained male runners compared to untrained counterparts, but there is no difference between well trained and untrained female participants (Magnusson et al. 2007), it therefore is possible that a region-specific hypertrophy, if present, may have been in part concealed owing to a gender effect.

Two weeks of detraining resulted in a reduction in tendon stiffness in MTCS (−12% change) and MTCX (−7% change) groups compared to week 8, with absolute values remaining superior to baseline measures. Interestingly, the MTCL group relative stiffness increased further by 11% compared to week 8 following 2 weeks detraining. This is reflected in Figure 5, where at week 10, elongation of the tendon is virtually identical to that at week 8 (although the force through the tendon is actually slightly higher), therefore reflecting a stiffer tendon. Following a further 2 weeks of detraining (week 12), MTCL stiffness had reduced (−19% change) which was the greatest relative loss during the detraining out of any other training groups, whereas MTCS and MTCX displayed identical relative reductions in stiffness (−11% change) between weeks 10–12. By week 12, the magnitude of increases in tendon stiffness relative to baseline was very similar between MTCL (35 ± 14%) and MTCX (32 ± 11%) groups, which again highlights the effectiveness of chronic loading in a lengthened condition.

Our current data on the effect of a relatively short period (14 days) of detraining on tendon properties are in support of the findings of De Boer et al. (2007) who showed that during 23 days of unilateral lower limb suspension in younger men, stiffness and Young's modulus decreased significantly after only 14 days, and accelerated during the next 9 days, hence describing a temporal pattern to losses in tendon mechanical properties. However, this temporal response only appeared in the MTCL and MTCX groups (who displayed much greater relative increments in tendon stiffness following training compared to MTCS). From days 14–28 (i.e., weeks 10–12) both of these groups experienced larger decrements in tendon stiffness than the initial 14 days of detraining (i.e., weeks 8–10). MTCX showed an initial −7% reduction in stiffness, followed by a greater −11% reduction in the second period of detraining, whereas MTCL, as mentioned previously, actually increased stiffness by +11% in the initial period of detraining, then experienced a large reduction (−19%) in the following 14 days. Kubo et al. (2010) reported a linear decrease in tendon properties (as seen in MTCS) following 3 months of training and proceeded by three further months of subsequent detraining (tendon properties measured every month). Our findings are in agreement with this study in that tendon properties are still enhanced compared to baseline following 1 month of detraining, however, because tendon properties were only assessed following 1 month detraining, it is still possible that a temporal pattern would have eventually formed into a linear pattern following a longer detraining period. It is suggested from the current results that tendon properties may be modulated depending on the degree of the initial adaptations to resistance training that is greater increases are eventually lost more rapidly, the longer the detraining carries on. This research is the first study to the authors' knowledge to demonstrate such a temporal response to detraining. It supports in part, the observations of De Boer et al. (2007) that involved complete unloading, although unloading is not necessarily comparable to detraining, where in the latter habitual activity is allowed to continue and would thus affect the rate/intensity of change in tendon properties. Notwithstanding our conclusions, future work, either using a long probe (Hansen et al. 2006) or the “stitching” technique (Onambele et al. 2007) could follow from this study to provide an impression of the total magnitude of the changes along the tendon.

Maximal torque increased significantly at all angles (except 60^o^) in MTCL, but only at four of the seven angles measured in MTCS. Previous data from studies involving isometric training have indicated that strength training adaptations are joint angle-specific (Thepaut-Mathieu et al. 1988; Kitai and Sale 1989; Kubo et al. 2006a), and that training at a more flexed knee angle (100^o^) results in significant changes in MVC over a greater range of angles (40–110^o^ of knee flexion) compared to a more extended knee angle that is, 50^o^ (40–80°). What is also interesting from the data in Figure 5 is that the MTCL group increased strength significantly at 30^o^ of knee flexion, despite not encountering this specific joint angle during the training excursion (i.e., 40–90^o^). This is in contrast to MTCS which did not increase torque levels at this angle despite dynamically passing through the joint angle during the training excursion (i.e., 0–50^o^). Furthermore, MTCX group had also significantly increased MVC at 30^o^ following training, again only passing dynamically through the joint angle. This study partly reaffirms the consensus from isometric training studies, that there is a less marked effect of joint angle specificity when training occurs with the muscle (or MTC) in a relatively lengthened position compared to a relatively shortened position (Thepaut-Mathieu et al. 1988; Kubo et al. 2006a). The phenomena that govern the adaptations to training angles not encountered, or those for no adaptation despite passing through an angle during loading, are as yet unexplained. Another relationship that was evident following training was the change in the torque-angle relationship in MTCS, but not in MTCL or MTCX. Although the mechanisms for such observations remain unclear, we propose below, an argument for the likely modulators of the observed changes.

Concentric training has also been purported to alter the operating range of muscle (e.g., Lynn et al. 1998), with a shift to shorter muscle (or sarcomere) lengths on the length–tension relationship of muscle. Conversely, an increase in tendon stiffness would decrease sarcomere shortening, shifting the length–tension relationship to longer muscle/sarcomere lengths. The VL muscle has been shown to operate over the ascending (<70°), plateau region (70°), and descending limbs of the length–tension relationship (Ichinose et al. 1997). In the MTCL and MTCX, peak torque occurred at 70^o^ (plateau region) before training, therefore an increase in K would have theoretically resulted in a shift toward the descending limb (>70^o^). However, in the same group of subjects, we observed significant increases in fascicle length (possibly indicating serial sarcomerogenesis in MTCL and MTCX- data under review elsewhere). This may explain why there was no change in angle of peak torque following training, as the two adaptations to training may have mitigated each other allowing the muscle to operate over the same portion of the length–tension relationship (Reeves et al. 2004). In MTCS, the optimal angle of torque production was 75^o^ pretraining and 70^o^ posttraining. Due to training at a shorter muscle length for an extended period, the muscle may have adapted in such a way as to produce higher forces (and keep individual sarcomeres operating around the plateau region) closer to the angle at which the muscle has been routinely operating. This would presumably have occurred despite increases in tendon stiffness, which would result in a shift towards longer muscle lengths. Whatever the case may be in terms of alterations to the working range of the sarcomere (which was beyond the scope of this study to determine), the current data imply that it is important to take account of the fact that dynamic resistance training kinematics bring about specific adaptations that could influence the joint torque-angle relationship.

## Conclusions

Training with the MTC in a lengthened position appears to enhance the improvements in the mechanical properties of the muscle–tendon unit to a greater degree than training in a shortened position. Interestingly, the adaptations to lengthened-position resistance loading are in fact comparable to those where the entire range-of-motion (and in fact higher loads) is used. The effects we describe here are associated with adaptations to both tensile properties of the in-series tendon and the torque-angle relationship. We propose that such adaptations result from a difference in the magnitude of mechanical stretch owing to the relative difference in length of the MTC. Nevertheless, the underlying mechanisms through which the mechanical transduction mediated these effects are as yet not known, other than a strong indication for no involvement of type I collagen synthesis change (as indicated through TGF-β1 data). The practical application of such findings may be of use to optimizing the training protocols of not only those in the athletic/sporting field but also to functionally challenged clinical populations.
